# A Pilot Study on Factors Involved with Work Participation in the Early Stages of Multiple Sclerosis

**DOI:** 10.1371/journal.pone.0105673

**Published:** 2014-08-25

**Authors:** Karin Van der Hiele, Huub A. M. Middelkoop, Rob Ruimschotel, Noëlle G. A. Kamminga, Leo H. Visser

**Affiliations:** 1 National Multiple Sclerosis Foundation, Maassluis, The Netherlands; 2 Leiden University, Department of Psychology, Section Health, Medical and Neuropsychology, Leiden, The Netherlands; 3 Medical Psychiatric Centre PsyToBe, Rotterdam, The Netherlands; 4 Leiden University Medical Centre, Department of Neurology, Leiden, The Netherlands; 5 St. Elisabeth Hospital, Department of Neurology, Tilburg, The Netherlands; 6 University of Humanistic Studies, Utrecht, The Netherlands; Charité University Medicine Berlin, Germany

## Abstract

**Background:**

Up to 30% of recently diagnosed MS patients lose their jobs in the first four years after diagnosis. Taking into account the personal and socio-economic importance of sustaining employment, it is of the utmost importance to examine factors involved with work participation.

**Objective:**

To investigate differences in self-reported functioning in recently diagnosed MS patients with and without a paid job.

**Methods:**

Self-reports of physical and cognitive functioning, depression, anxiety and fatigue were gathered from 44 relapsing-remitting MS patients diagnosed within 3 years.

**Results:**

Patients with a paid job (57%) reported better physical functioning (p<0.001), better memory functioning (p = 0.01) and a lower physical impact of fatigue (p = 0.018) than patients without a paid job. Physical functioning was the main predictor of employment status in a logistic regression model. In those with a paid job better memory functioning (r = 0.54, p = 0.005) and a lower social impact of fatigue (r = −0.46, p = 0.029) correlated with an increased number of working hours.

**Conclusion:**

Better physical functioning is the primary factor involved with increased work participation in early MS. Better self-reported memory functioning and less social fatigue were associated with increased working hours. These findings highlight the importance of battling these symptoms in the early stages of MS.

## Introduction

Multiple Sclerosis (MS) is the most common cause of neurological disability in young and middle-aged adults [1] [2]. Despite a recent work history, 70 to 80% of people with MS are unable to retain employment following diagnosis [3]. MS patients may be particularly vulnerable for job loss during the first years after diagnosis. A Danish study reported that the number of MS patients receiving disablement pension increased from 4% to 30% in the first four years after diagnosis [4]. Patients with a recent diagnosis of MS report a substantially lower health-related quality of life than healthy controls [5]. Work participation is very important in this context, leading to financial security, social contacts and providing a feeling of usefulness and satisfaction. In a large cohort of relapsing-remitting MS patients in the early stages of the disease, it was found that work participation and a higher educational level were both associated with a higher health-related quality of life, independent of sex, age, disability level and disease duration [6]. These findings underscore the importance of maintaining a job after a recent diagnosis of MS.

The causes of unemployment in MS involve a complex interaction between disease-related factors, the working environment, job demands and personal factors [7]. Many studies on work ability or work status in MS focus on the importance of physical and cognitive abilities in maintaining a job. Physical disabilities, including decreased mobility, impaired hand function, visual impairments, fatigue and pain have been associated with job loss or changes at work in patients with MS [8]. Cognitive abilities were found to be equally or even more important in maintaining a job. Several studies identified working memory, episodic memory and mental speed as important predictors of employment status [9] [10]. In addition to cognitive and physical problems, more depression and lower scores on the personality scale ‘agreeableness’ have been associated with an increased likelihood of job loss, reduced working hours, or changing the type of job [8] [11]. Many of the above mentioned ‘risk factors’ for job loss or reduced working hours may already be present in recently diagnosed MS patients [4] [9] [10] [12] [13]. Reduced mental speed, disturbed non-verbal memory and impaired executive functioning have been observed during the early stages of MS [4] [13]. In comparison with controls, a higher incidence of fatigue, depression and apathy were observed in recently diagnosed MS patients [12].

Taking into account the personal and socio-economic importance of sustaining employment, especially in patients with a recent diagnosis of MS, it is of the utmost importance to examine which factors are involved with work participation. Few studies have focussed on work participation in patients with a relatively recent diagnosis of MS. The current study is based on data collected for a large community-based study on cognitive and psychological problems in patients with MS [14] [15]. In this pilot study on work participation in MS, we examined differences in self-reported physical abilities, cognitive functioning, depression, anxiety and fatigue in recently diagnosed relapsing-remitting MS patients with and without paid employment. As a secondary objective, we examined relations between these factors and the number of working hours in employed patients.

## Materials and Methods

### Study population

The ‘MS Cognition Study’ provides an inventory of cognitive and psychological problems in a community-based cohort of Dutch MS patients [15]. In short, questionnaires about demographics, physical functioning, cognitive abilities, mood and fatigue were completed by 718 MS patients who were 18 years or older. No other inclusion or exclusion criteria were applied.

For the current study, we selected all patients between 18 and 64 years old and diagnosed with relapsing-remitting MS (RRMS) no more than three years ago. Based on these criteria we included 44 patients.

### Ethics statement

The study was approved by the Medical Ethical Committee of the St. Elisabeth Hospital in Tilburg, The Netherlands. All subjects provided written informed consent.

### Demographics and disease characteristics

A general questionnaire was used to obtain information on demographic characteristics, including educational level and employment status, number of working hours, time since diagnosis, MS subtype, and pharmacological treatment. The employed group consisted of patients who had a paid job, either full-time (35 or more hours per week), part-time (12–34 hours per week) or less than 12 hours a week. One participant indicated being a student and having a paid job. She was included in the employed group. The unemployed group consisted of patients without a paid job, including homemakers, volunteers, patients receiving disability or unemployment benefits, patients on prolonged medical leave, or early retirement.

### Physical functioning

The physical component of the Short Form-36 Health Survey (SF-36) provided a measure of physical functioning. This questionnaire inquires into limitations in daily activities, e.g. being able to run, move a table, climb stairs, lift or carry groceries, walk a certain distance, wash and dress. Possible scaled scores range from 0 to 100, with higher scores indicative of better physical functioning. Based on normative research in a Dutch-speaking sample of adults, scores at or below 60 are indicative of below average physical functioning [16]. The SF-36 is considered a sensitive measure of physical functioning in newly diagnosed MS patients [17].

### Cognitive abilities

A general rating of cognitive functioning was obtained using the scale ‘insufficiency of thinking and acting’ of the Symptom Checklist-90-R (SCL-90-R) [18]. Possible scores range from 9–45. Scores at or above 15 are indicative of insufficient thinking and acting.

Self-ratings of executive functioning were obtained using the Behavioural Assessment of the Dysexecutive Syndrome-Dysexecutive questionnaire (BADS DEX) [19]. This questionnaire examines behavioural, cognitive and emotional components of executive functioning. BADS DEX self-report scores higher than 37 are considered suggestive of executive impairment.

The Disability and Impact Profile (DIP) [20] provided self-ratings of memory and concentration. Possible scores range from 0 (bad) to 10 (good).

### Depression and anxiety

Self-report measures of anxiety and depression were obtained using the Hospital Anxiety and Depression Scale (HADS) [21]. The HADS provides valid markers of major depression and generalized anxiety disorder in MS [22]. Possible scores per domain, i.e. anxiety or depression, range from 0–21 and scores at or above 8 can be considered indicative of major depression or generalized anxiety disorder.

### Fatigue

The Fatigue Impact Scale (FIS) [23] was used to assess the impact of fatigue on daily functioning in physical, cognitive, and social dimensions. Possible scores on the physical and cognitive dimensions both range from 0–40, with higher scores indicative of increased fatigue impact. Possible scores on the social dimension range from 0–80, with higher scores indicative of increased fatigue impact.

### Statistical analysis

SPSS for Windows (release 21.0) was used for data analysis. In case of missing items in a questionnaire (with a maximum of 5%) a proportionate score was calculated for that respondent by averaging the remaining items. We examined differences in demographic characteristics, physical functioning, cognitive abilities, depression, anxiety and fatigue between employed and unemployed patients using parametric and non-parametric tests where appropriate. A logistic regression analysis was used to examine predictors of employment status (employed/not employed). As possible predictors we included characteristics that differed significantly between employed and unemployed MS patients. Pearson's correlation analyses were used to examine correlations between the number of working hours and physical functioning, cognitive abilities, depression, anxiety and fatigue in employed MS patients. The level of statistical significance was set at p≤0.05.

## Results

### Demographic and disease characteristics of the study sample

Table 1 lists the demographic and disease characteristics for employed and unemployed patients. The study sample included 44 recently diagnosed patients with RRMS of which 57% (N = 25) had a paid job (28% working full-time, 64% working part-time, and 8% working less than 12 hours per week). Of those with a paid job 68% (N = 17) performed white collar work (professional, administrative work or management) and 32% (N = 8) blue collar work (skilled manual labour). In patients with a paid job 20% also performed unpaid work outside their homes, e.g. by doing volunteer work or following an internship. In those without a paid job, 11% performed unpaid work.

**Table 1:** Demographic and disease characteristics of the study sample.

	Patients with paid employment (N = 25)	Patients without paid employment (N = 19)
working hours per week	25.8 (±10.7)	-
full-time workers	28% (N = 7)	-
part-time workers	64% (N = 16)	-
working less than 12 hours per week	8% (N = 2)	-
white collar work	68% (N = 17)	-
blue collar work	32% (N = 8)	-
unpaid work outside home	20% (N = 5)	11% (N = 2)
sex (female)	88% (N = 22)	90% (N = 17)
age (years)	35.6 (±7.1)	38.8 (±8.8)
time since diagnosis (years)	2.0 (±0.9)	2.0 (±0.7)
educational level^a^	5.6 (±1.0)	5.7 (±0.7)
(1) <primary education	0% (N = 0)	0% (N = 0)
(2) primary education	4% (N = 1)	0% (N = 0)
(3) <low level secondary education	0% (N = 0)	0% (N = 0)
(4) low level secondary education	4% (N = 1)	0% (N = 0)
(5) average level secondary education	32% (N = 8)	36.8% (N = 7)
(6) high level secondary education	48% (N = 12)	52.6% (N = 10)
(7) university degree	12% (N = 3)	10.5% (N = 2)
use of immunomodulators	92% (N = 23)	79% (N = 15)
use of antidepressants	8% (N = 2)	11% (N = 2)
use of benzodiazepines	0	11% (N = 2)

Percentages (N) or means (± SD) are reported. ^a^educational level: (1) less than six years of primary education; (2) finished six years of primary education; (3) six years primary education and less than two years of low level secondary education; (4) four years of low level secondary education; (5) four years of average level secondary education; (6) five years of high level secondary education; (7) university degree. No significant group differences were found at p≤0.05.

No differences in demographic or disease characteristics were found between employed and unemployed patients. Relatively few male MS patients were included in this study (N = 5). A similar number of males and females had a paid job (respectively 60% and 56%). The number of working hours was higher in the male participants; on average 40.3 working hours in males versus 23.9 working hours in females. Although male/female differences are of interest in the context of work participation, the low number of male participants impeded the inclusion of this variable in further analyses.

### Physical functioning, cognitive abilities, mood and fatigue

Self-reported physical functioning, cognitive abilities, mood and fatigue are listed in Table 2. Unemployed patients experienced more physical disabilities (t(28) = −4.42, p<0.001), reported worse memory functioning (t(42) = −2.69, p = 0.01) and a higher physical impact of fatigue (t(36) = 2.49, p = 0.018) than employed patients.

**Table 2:** Differences in self-reported functioning between patients with and without paid employment.

	Patients with paid employment (N = 25)	Patients without paid employment (N = 19)	p
**Physical abilities**			
physical functioning	77.4 (±14.2)	50.8 (±23.1)	p<0.001
**Cognitive abilities**			
insufficiency of thinking and acting	18.2 (±5.4)	19.8 (±5.5)	n.s.
executive functioning	24.1 (±8.7)	26.7 (±13.0)	n.s.
memory	7.4 (±1.6)	5.8 (±2.4)	p = 0.01
concentration	7.3 (±1.5)	6.1 (±2.4)	n.s.
**Mood**			
depression	4.5 (±3.3)	5.7 (±4.7)	n.s.
anxiety	6.6 (±3.5)	8.4 (±3.0)	n.s.
**Fatigue**			
physical impact of fatigue	18.0 (±8.8)	25.2 (±8.9)	p = 0.018
cognitive impact of fatigue	17.1 (±10.1)	21.7 (±10.9)	n.s.
social impact of fatigue	27.2 (±15.9)	37.9 (±18.4)	n.s.

Means (± SD) are reported. Mann-Whitney U and independent t-tests were used to examine group differences.

### Logistic regression model of employment status

A logistic regression analysis was conducted to identify independent predictors of employment status. Self-rated measures of physical functioning, memory and the physical impact of fatigue were entered as covariates. In the resulting regression model (Table 3) physical functioning was significantly associated with employment status in early MS, while self-ratings of memory and the physical impact of fatigue were not. Better physical functioning was associated with increased odds of being employed. The model correctly classifies 73.7% of the cases, which is significantly higher than the 55.3% classification accuracy when only the constant is included in the regression model (Hosmer & Lemeshow χ^2^(3) = 15.9, p = 0.001).

**Table 3:** Logistic regression model of employment status.

Included	B (SE)	exp (B)	Wald	P value
Constant	−5.14 (3.3)	0.006	2.396	0.12
Physical functioning	0.07 (0.03)	1.07	5.13	0.02
Physical impact of fatigue	−0.003 (0.06)	0.997	0.003	0.96
Memory	0.15 (0.25)	1.16	0.38	0.54

The logistic regression model included employment status (paid job/no paid job) as dependent variable and physical functioning, physical impact of fatigue and memory as covariates. R^2^ = 0.34 (Cox & Snell), 0.46 (Nagelkerke).

### Relations between self-reported functioning and number of working hours

In the employed patients we found that better memory functioning (r = 0.54, p = 0.005) and a lower social impact of fatigue (r = −0.46, p = 0.029) correlated with an increased number of working hours. The other variables, i.e. physical functioning, insufficiency of thinking and acting, executive functioning, concentration, fatigue, depression and anxiety, were unrelated to the number of working hours.

When looking specifically at patients with a white or blue collar job, we found similar relations in patients with a white collar job; better memory functioning (r = 0.70, p = 0.003) and a lower social impact of fatigue (r = −0.54, p = 0.045) were correlated with an increased number of working hours. However, no relations were found in patients with a blue collar job.

## Discussion

In the early stages of MS, unemployment rates rise dramatically despite the fact that many patients have a recent working history [4]. Few studies have focussed on factors involved with unemployment in the first years after being diagnosed with an unpredictable, chronic disease. In the current study we found that self-perceived better physical functioning is the primary factor involved with work participation in recently diagnosed MS patients. In addition to better physical functioning, MS patients with a paid job reported better memory abilities and a lower physical impact of fatigue than patients without a paid job. We found that particularly in patients with a ‘white collar’ type of job, more favourable memory ratings and less social fatigue are related to an increased number of working hours.

Only 57% of the recently diagnosed MS patients had a paid job. In the total Dutch population in 2010, labour participation in similar age groups varied from 66% (15–39 years old) to 80% (40–54 years old) [24]. Our finding is compatible with a 4-year Danish follow-up study that found an employment rate of 55% at approximately 3 years after diagnosis [4]. As opposed to the Danish sample which included 73% full-timers, the majority, i.e. 64% of the MS patients in the current study worked part-time. Work participation is considered high in The Netherlands as compared with other European countries, and so is the number of people working on a part-time basis [25]. The relatively high number of female participants may have inflated the percentage of part-time workers in this study. A common finding is that the high unemployment rate in the early stages of MS is alarming.

### Factors involved with work participation

Important differences were noted between recently diagnosed MS patients with and without a paid job.

Higher physical functioning was particularly associated with having a paid job, as this was the only significant predictor of employment status. Many other studies have recognized the hindrance of physical limitations in maintaining a job in MS patients [26]. One of the few studies in recently diagnosed MS patients found that both physical functioning and cognitive status predicted the patient's vocational status seven years after diagnosis [27]. It has been suggested that many patients with MS stop working before the physical limitations become significant [7]. We found that physical functioning was not related to the number of working hours, even in more physically demanding jobs. This may suggest that when the perceived physical limitations pass a certain threshold, the arising problems are not dealt with by cutting back on the number of working hours, but by leaving the workplace. Workplace-factors are important in this respect, including the (presumed) level of flexibility and support available from employers and colleagues. Especially in the early stages of MS vocational rehabilitation is possible [7].

We discovered that patients without a paid job rated their memory abilities lower than patients with a paid job. It seems intuitive that being able to encode and retrieve information, e.g. remembering appointments, to-do lists, and client information, are important qualities at work. Although cognitive symptoms were not reported as a reason to cut back hours in the study by Smith and Arnett (2005) [28], better memory functioning was strongly related to more working hours in our sample. This was specifically true in patients with a white collar job, performing skilled professional work where memory problems may be perceived as more interfering. In previous studies, working memory, episodic memory and mental speed have been identified as important predictors of employment status [9] [27] [29]. Although we used solely self-report measures our results concerning memory abilities are in line with these findings. In this context it should be noted that self-report and objective measures of cognitive functioning may not always correspond, but can be viewed as complementary [14] [30]. In the early stages of MS perceived forgetfulness in particular may signal early problems at work.

Recently diagnosed patients without a paid job reported more physical fatigue than patients with a paid job. Physical fatigue was not an independent predictor in our model of employment status, which may be explained by the close relation between physical fatigue and physical functioning. Fatigue tends to exacerbate other symptoms of MS, including physical disability [31]. The observed influence of physical fatigue on employment is consistent with other studies using an objective measure of fatigue [28] [32] [33]. A qualitative study reported that fatigue was the most frequently mentioned symptom (reported by 69.5%) related to employment loss in patients with MS [34]. A new finding is that physical fatigue is already an important obstacle for work participation in the early stages of MS. Upon inspection of our data, this is particularly the case in patients with a more physically demanding job. In a follow-up study of MS patients working full-time, fatigue was mentioned as a primary symptom for cutting back on the number of working hours [28]. In the current study, we found relations between experiencing more social fatigue and working less hours per week, specifically in white collar workers. Although we are unaware of patients' motivations to work full-time, part-time or less hours, these are very interesting findings. It may be that social fatigue can be dealt with by working less hours, while the experienced physical fatigue was severe enough to stop working and does not even disappear when leaving employment. By monitoring fatigue severity and impact -in all its forms- from the early stages of MS and offering timely interventions, its impact on work participation may be reduced.

### Strengths and limitations

Strengths of the current study include its focus on patients who were recently diagnosed. This is a particularly vulnerable group of MS patients at risk of job loss [3] [5] [12]. We included MS patients from a large community-based sample, which may be more representative of the general MS population than patients recruited in a hospital setting. A main limitation of this study is its cross-sectional design, which makes it impossible to make any causal inferences. The MS Cognition Study did not specifically focus on MS patients who were recently diagnosed, resulting in a limited study sample. Also, no information was available about co-morbidity, absence from work, previous work participation, or reasons to choose for the current work situation. A cross-sectional study, such as ours, would greatly benefit from a retrospective inventory of previous paid and unpaid work, even before receiving the diagnosis of MS. This may provide a more detailed view of the patients' work history if one exists. Longitudinal studies are needed to examine factors involved with work participation over the course of several years. Besides disease-related factors, these studies may also examine personal factors, including financial considerations, the patient's support system, coping abilities, and the effects of the working environment and job demands on work participation [7]. In a longitudinal design, work participation may be viewed in a broader manner including not only job loss but also changes in working hours, work responsibilities, and days absent from work [29].

We did not use a control group. Best practice in future research on work participation is to include control groups, preferably both healthy persons and patients with other chronic (neurological) diseases. The labour market, government stimulation policies for the chronically ill, and welfare benefits are dynamic, accentuating the necessity of monitoring work participation in other groups.

Another consideration is the use of self-report measures. Self-reported cognitive performance may not always correspond with actual cognitive performance [14] [35]. Other studies in patients with MS have shown self-reports of disability, depression, and anxiety to be reliable as compared with clinician-derived data [22] [36]. Self-report measures do provide us with a unique view on patients' perceptions and may identify problems in need of intervention.

We used questionnaires for physical functioning, cognitive abilities, mood and fatigue that have good psychometric properties and have been validated for use in Dutch populations. Future studies on work participation in MS may benefit from also including internationally well-known instruments that have been validated for use in MS populations.

### Clinical implications

Our findings highlight the importance of battling physical disabilities, perceived memory problems and fatigue in the early stages of MS. Further longitudinal studies are needed to examine factors involved with work participation in MS. At the same time there is a need for timely intervention and information provision for MS patients, preferably while people are still at work. Self management intervention programs may be of particular value in this respect. Ideally, health care professionals and employment specialists should work together in the effort to help MS patients maintain employment or re-enter the workplace [7]. This may include a perception-shift on the part of both professionals and people in the direct environment of the person with MS by seeing not only limitations but also possibilities.
